# Supplementary material for: Phenolic Compounds and Ginsenosides in Ginseng Shoots and Their Antioxidant and Anti-Inflammatory Capacities in LPS-Induced RAW264.7 Mouse Macrophages
Source: Int J Mol Sci. 2019 Jun 17;20(12):2951. doi: 10.3390/ijms20122951 (PMC6627944; doi:10.3390/ijms20122951)
Supplement: Supplementary file 1 [file ijms-20-02951-s001.pdf]

1. Precision test

The ginsenoside standard solution was injected at 20 μl for 6 times. The peak areas of Rg1, Re, Rf, S-Rg2, S-Rh1, R-Rg2, Rc, Rb2, Rb3, F1, Rd, F4, S-Rg3, R-Rg3, PPT, Rg5, CK, and Rh2 are shown in supplementary Table S1, and the RSD values are 0.27%, 0.93%, 0.53%, 0.60%, 0.78%, 0.63%, 0.53%, 1.37%, 0.55%, 0.99%, 0.61%, 1.21%, 1.66%, 0.66%, 0.53%, 0.52%, 0.56%, and 0.89%, respectively. RSD values are all less than 5%.

Supplementary Table S1. Precision test of 18 pure ginsenoside.

	Peak area						STDEV	PAA	RSD
Rg1	4644530	4660481	4676673	4653753	4642498	4661444	12602.384	4656563.167	0.27
Re	3613610	3547624	3623084	3640988	3633536	3619843	33549.618	3613114.167	0.93
Rf	5146204	5069193	5132631	5135027	5120333	5121514	27024.996	5120817.000	0.53
S-Rg2	5466383	5370023	5422177	5433850	5401283	5409531	32430.831	5417207.833	0.60
S-Rh1	5002115	5008690	5095992	5086336	5068083	5055814	39363.737	5052838.333	0.78
R-Rg2	4893735	4866024	4935120	4933233	4936443	4943954	31077.789	4918084.833	0.63
Rc	8624708	8497775	8607284	8605395	8586932	8596302	45204.375	8586399.333	0.53
Rb2	3474165	3549262	3596381	3600118	3589359	3591616	48987.121	3566816.833	1.37
Rb3	3859974	3803108	3848988	3857181	3832726	3837797	20946.155	3839962.333	0.55
F1	6053883	6117046	6200105	6216105	6171009	6185513	60974.853	6157276.833	0.99
Rd	4135670	4129339	4186630	4184478	4171377	4177768	25215.677	4164210.333	0.61
F4	6143709	5923155	6000872	6006879	5983301	5991797	72855.694	6008285.500	1.21
S-Rg3	4936409	4716321	4767063	4774332	4748343	4739845	79160.572	4780385.500	1.66
R-Rg3	2388000	2377950	2383343	2408697	2401323	2365100	15816.598	2387402.167	0.66
PPT	6854960	6785223	6874527	6883210	6861189	6875615	36070.769	6855787.333	0.53
Rg5	15428450	15348014	15545228	15560972	15492239	15513773	80208.138	15481446.000	0.52
CK	5952940	5923270	6001824	6007122	5987622	5997509	33200.490	5978381.167	0.56
Rh2	5211150	5066899	5132155	5145494	5134095	5139047	45853.738	5138140.000	0.89

PAA: peak area in average; STDEV: standard deviation; RSD: relative standard deviation; Rg1, ginsenoside-Rg1; Re, ginsenoside-Re, Rf, ginsenoside-Rf; S-Rg2, ginsenoside-Rg2(S-form); S-Rh1, ginsenoside-Rh1(S-form); R-Rg2, ginsenoside-Rg2(R-form); Rc, ginsenoside-Rc; Rb2, ginsenoside-Rb2; Rb3, ginsenoside-Rb3; F1, ginsenoside-F1; Rd, ginsenoside-Rd; F4, ginsenoside-F4; S-Rg3, ginsenoside-Rg3 (S-form); R-Rg3, ginsenoside-Rg3 (R-form); PPT, S-protopanaxatriol; Rg5, ginsenoside-Rg5; CK, ginsenoside-compound K; Rh2, ginsenoside-Rh2.

2. Stability test

Ginsenoside standard solution was injected at 0, 3, 6, 9, 12 and 24 h respectively. The peak areas of Rg1, Re, Rf, S-Rg2, S-Rh1, R-Rg2, Rc, Rb2, Rb3, F1, Rd, F4, S-Rg3, R-Rg3, PPT, Rg5, CK, and Rh2 are shown in supplementary Table S2, RSD values are 1.75%, 0.70%, 0.32%, 0.44%, 0.24%, 0.27%, 0.27%, 0.28%, 0.32%, 0.25%, 0.38%, 0.32%, 0.32%, 0.30%, 0.30%, 0.27%, 0.34%, and 1.19%, respectively, and the RSD values are all less than 5%, indicating that the standard solution was stable within 24 hours.

Supplementary Table S2. Stability test of 18 pure ginsenoside.

	Peak area						STDEV	PAA	RSD
Rg1	4345666	4378391	4438630	4513571	4545872	4413705	77468.132	4439305.833	1.75
Re	3672157	3654257	3615864	3644085	3632440	3602484	25466.879	3636881.167	0.70
Rf	5044797	5025270	5055223	5037909	5049885	5013088	15913.492	5037695.333	0.32
S-Rg2	5216677	5227210	5242602	5247775	5283966	5238492	23080.063	5242787.000	0.44
S-Rh1	5077970	5083606	5089306	5087335	5086675	5057204	12008.406	5080349.333	0.24
R-Rg2	4875288	4874222	4894256	4894130	4903330	4873316	13027.549	4885757.000	0.27
Rc	8456270	8447329	8473738	8469119	8497612	8430388	23251.063	8462409.333	0.27
Rb2	3430936	3426315	3443495	3439400	3449205	3426568	9492.227	3435986.500	0.28
Rb3	3744569	3720809	3734715	3731144	3748457	3719386	11940.801	3733180.000	0.32
F1	6076507	6071973	6087379	6091463	6110319	6071255	14960.197	6084816.000	0.25

Rd	4080439	4039975	4049161	4058210	4068331	4043361	15591.692	4056579.500	0.38
F4	5857145	5871457	5887320	5888107	5911479	5870524	18917.175	5881005.333	0.32
S-Rg3	4731412	4744318	4748493	4758117	4774441	4740393	15061.750	4749529.000	0.32
R-Rg3	2418342	2415588	2423471	2421453	2433515	2412643	7334.409	2420835.333	0.30
PPT	6796782	6812676	6791036	6809018	6847559	6799799	20275.997	6809478.333	0.30
Rg5	15198740	15225448	15252977	15263057	15305020	15196137	41869.852	15240229.83	0.27
CK	5961209	5971227	6000932	5993299	6011357	5969359	20082.829	5984563.833	0.34
Rh2	5027801	5036052	5145374	5052590	5162969	5036948	60601.901	5076955.667	1.19

PAA: peak area in average; STDEV: standard deviation; RSD: relative standard deviation; Rg1, ginsenoside-Rg1; Re, ginsenoside-Re, Rf, ginsenoside-Rf; S-Rg2, ginsenoside-Rg2(S-form); S-Rh1, ginsenoside-Rh1(S-form); R-Rg2, ginsenoside-Rg2(R-form); Rc, ginsenoside-Rc; Rb2, ginsenoside-Rb2; Rb3, ginsenoside-Rb3; F1, ginsenoside-F1; Rd, ginsenoside-Rd; F4, ginsenoside-F4; S-Rg3, ginsenoside-Rg3 (S-form); R-Rg3, ginsenoside-Rg3 (R-form); PPT, S-protopanaxatriol; Rg5, ginsenoside-Rg5; CK, ginsenoside-compound K; Rh2, ginsenoside-Rh2.

3. Repeatability test

Six samples of GSE6 were prepared at a concentration of 10 mg/mL for HPLC analysis according to the method mentioned in section 3.2. The peak areas of Rg1, Re, S-Rg2, S-Rh1, R-Rg2, Rc, Rd, F4, S-Rg3, R-Rg3, PPT, Rg5, CK, and Rh2 are shown in supplementary Table S2, RSD values are 1.95%, 1.73%, 3.04%, 3.15%, 3.93%, 1.89%, 0.78%, 2.00%, 4.17%, 1.56%, 2.43%, 2.83%, and 2.45%, respectively, and the RSD values are all less than 5%, indicating that the standard solution was with good repeatability.

Supplementary Table S3. Repeatability of GSE6.

	Peak area						STDEV	PAA	RSD
Rg1	75449	76568	74692	78912	75804	75427	1487.454	76142.000	1.95
Re	74839	73597	76515	72976	75487	74229	1287.776	74607.167	1.73
S-Rg2	2323439	2459521	2392537	2295478	2469129	2437918	72829.896	2396337.000	3.04
S-Rh1	1875348	1875348	1916234	1799213	1837586	1963689	57721.982	1877903.000	3.07
R-Rg2	1246915	1287932	1305261	1298102	1349865	1358121	41240.332	1307699.333	3.15
Rc	1004071	1018145	1103211	1093827	1076592	1035987	41499.492	1055305.500	3.93
Rd	442418	447576	437482	447385	427477	449477	8336.599	441969.167	1.89
F4	8390568	8355771	8329288	8235474	8335474	8427595	65337.660	8345695.000	0.78
S-Rg3	1806700	1837359	1877725	1897245	1887245	1824851	37131.259	1855187.500	2.00
R-Rg3	991919	1012919	1013892	995680	1102684	993278	42432.800	1018395.333	4.17
PPT	1785015	1742725	1781810	1760598	1710598	1754096	27459.559	1755807.000	1.56
Rg5	4885949	4723208	4772363	4648519	4548519	4685254	114444.410	4710635.333	2.43
CK	56054	60362	56937	56547	56216	56599	1618.480	57119.167	2.83
Rh2	2266174	2281432	2148764	2259874	2178543	2259647	54658.204	2232405.667	2.45

PAA: peak area in average; STDEV: standard deviation; RSD: relative standard deviation; Rg1, ginsenoside-Rg1; Re, ginsenoside-Re, Rf, ginsenoside-Rf; S-Rg2, ginsenoside-Rg2(S-form); S-Rh1, ginsenoside-Rh1(S-form); R-Rg2, ginsenoside-Rg2(R-form); Rc, ginsenoside-Rc; Rb2, ginsenoside-Rb2; Rb3, ginsenoside-Rb3; F1, ginsenoside-F1; Rd, ginsenoside-Rd; F4, ginsenoside-F4; S-Rg3, ginsenoside-Rg3 (S-form); R-Rg3, ginsenoside-Rg3 (R-form); PPT, S-protopanaxatriol; Rg5, ginsenoside-Rg5; CK, ginsenoside-compound K; Rh2, ginsenoside-Rh2.
